# Polydatin has anti‐inflammatory and antioxidant effects in LPS‐induced macrophages and improves DSS‐induced mice colitis

**DOI:** 10.1002/iid3.455

**Published:** 2021-05-19

**Authors:** Guangxin Chen, Ziyue Yang, Da Wen, Jian Guo, Qiuhong Xiong, Ping Li, Liping Zhao, Junping Wang, Changxin Wu, Lina Dong

**Affiliations:** ^1^ Institute of Biomedical Sciences Shanxi University Taiyuan China; ^2^ Key Laboratory of Chemical Biology and Molecular Engineering of Ministry of Education Shanxi University Taiyuan Shanxi China; ^3^ Department of General Surgery, Shanxi Provincial People's Hospital Affiliate of Shanxi Medical University Taiyuan Shanxi China; ^4^ Department of Pathology, Shanxi Provincial People's Hospital Affiliate of Shanxi Medical University Taiyuan Shanxi China; ^5^ Department of Gastroenterology, Shanxi Provincial People's Hospital Affiliate of Shanxi Medical University Taiyuan Shanxi China; ^6^ Central Laboratory, Shanxi Provincial People's Hospital Affiliate of Shanxi Medical University Taiyuan Shanxi China

**Keywords:** anti‐inflammatory, antioxidant, IBD, intestinal epithelial barrier, polydatin

## Abstract

Polydatin (PD), a monocrystalline compound isolated from the root and rhizome of *Polygonum cuspidatum*, is widely used in inhibiting the inflammatory response and oxidative stress. PD has an anti‐inflammatory effect on colitis mice; however, information regulating the mechanism by which maintains the intestinal epithelium barrier is currently scarce. Here, we assessed the anti‐inflammatory and antioxidant of PD in lipopolysaccharide (LPS)‐induced macrophages in vitro, and explored its effects on inhibiting intestinal inflammation and maintaining the intestinal epithelium barrier in dextran sodium sulfate (DSS)‐induced colitis mice. Results showed that PD reduced the level of proinflammatory cytokines and enzymes, including tumor necrosis factor‐α, interleukin‐4 (IL‐4), IL‐6, cyclooxygenase‐2, and inducible nitric oxide synthase, in LPS‐induced macrophages, and improved the expression level of IL‐10. PD maintained the expression of tight junction proteins in medium (LPS‐induced macrophages medium)‐induced MCEC cells. Additionally, PD inhibited the phosphorylation of nuclear factor‐κB (NF‐κB), p65, extracellular signal‐regulated kinase‐1/2, c‐Jun N‐terminal kinase, and p38 signaling pathways in LPS‐induced macrophages and facilitated the phosphorylation of AKT and the nuclear translocation of Nrf2, improving the expression of HO‐1 and NQO1. Furthermore, PD ameliorated the intestinal inflammatory response and improved the dysfunction of the colon epithelium barrier in DSS‐induced colitis mice. Taken together, our results indicated that PD inhibited inflammation and oxidative stress, maintained the intestinal epithelium barrier, and the protective role of PD was associated with the NF‐κB p65, itogen‐activated protein kinases, and AKT/Nrf2/HO‐1/NQO1 signaling pathway.

## 1 INTRODUCTION

The mammalian gut is a highly immune‐active ecosystem that is the site of digestion, absorption, and assimilation of nutrients. It harbors commensal microorganisms, while excluding or eliminating ingested pathogens. Disruption of the intestinal immune system balance leads to conditions, such as inflammatory bowel diseases (IBDs) and gastrointestinal infections.^1^ IBDs are typically classified as either Ulcerative colitis or Crohn's disease^2^ and are generally characterized by chronic, progressive, and relapsing inflammation which is associated with the dysregulation of intestinal mucosal immune system and commensal ecosystem.^3^ Thus far, the etiopathogenesis underlying the disease remains incompletely understood; however, genetic predisposition, immune response, environmental triggers, and the gut microbiome may implicate the morbidity of IBDs.^4^ IBDs began to emerge in Western countries during the middle of the 20th century; however, it has become a global disease since entry into the 21st century.^5^ Although monoclonal antibodies, including antitumor necrosis factor (anti‐TNF), and interleukin‐12 (IL‐12)/IL‐23 antibody, have been used to treat patients with IBDs and have achieved beneficial functions in most patients, some individuals remain unresponsive or lose response to therapies over time.^6^ Therefore, the exploration of new drugs to treat IBDs remains necessary.

Over time, there have been considerable therapeutic advances in the treatment for IBDs. These include traditional general anti‐inflammatory and anti‐immunosuppressive drugs, such as 5‐aminosalicylates, corticosteroids, and thiopurines, biologic therapies, such as anti‐TNF‐α and anti‐IL‐12/23 antibody, and integrin to reduce inflammatory cells\migration.^7^ In recent years, some small molecules that inhibit the Janus kinase (JAK) signaling pathway have been used clinically.^8^ Recently developed drugs have been successful in the treatment of IBDs, and an increasing number of new drugs are on the horizon. All approaches face a common challenge—complete mucosal healing, the coveted therapy target of prognosis.^9^ However, most of the present therapies focus on anti‐inflammatory and no treatments currently wholly focus on complete resolution in IBDs.^7^

PD is isolated from the *Polygonum cuspidatum*, a member of the Polygonaceae family that is widely used as foodstuffs and medicine in Asian.^10^ PD has different biological effects in a variety of diseases, including cancer,^11^ ischemia‐reperfusion injury,^12^ acute kidney injury,^13^ nonalcoholic steatohepatitis,^14^ ethanol‐induced liver injury,^15^ Parkinson's disease,^16^ and atherosclerosis.^17^ The versatile compound also shows potential functions as an antioxidant,^18^ anti‐inflammatory,^19^ and in the improvement of apoptosis.^20^

Although PD has a potential protective role in dextran sodium sulfate (DSS)‐induced mice,^21^ information regarding the maintenance of epithelial barrier of intestine in IBD is currently lacking. Therefore, in this study, we aimed to explore the effects of PD on the intestinal inflammation and intestinal epithelial barrier integrity in DSS‐induced mice.

## 2 MATERIALS AND METHODS

### 2.1 Animal model

In this study, we utilized 6‐to‐8‐week‐old C57BL/6 mice. All mice were obtained from the Laboratory Animal Center of Shanxi Provincial People's Hospital and housed at 22–23°C with a 12/12 h light/dark cycle and ad libitum access to food and water. All mice were divided into four groups and each group contain 14 mice: sham group; PD group, wherein PD was dissolved in sterile saline and administered via oral gavage once a day lasting for 11 days; DSS group, wherein DSS (5%, weight/volume, g/ml) was administered via drinking water for 6 days; DSS + PD group, PD was administered as a pretreatment for 5 days before before DSS treatment and finally for a further 6 days, followed by DSS treatment for 6 days. All experiments were approved by Shanxi Provincial People's Hospital Institutional Animal Care and Use Committee.

### 2.2 Disease activity index

Weight loss, stool characteristics, and fecal occult blood were recorded during the progression of the DSS‐induced mouse IBD model. The disease activity index (DAI) was calculated based on the scoring system as previously described.^22^

### 2.3 Hematoxylin and eosin (H&E) staining

The colon tissues of the experimental mice were collected and washed with cold phosphate‐buffered saline (PBS). The colonic segments were fixed using 4% paraformaldehyde, embedded in paraffin, and sectioned into 3 μM. The paraffin sections of colon tissue were performed with hematoxylin and eosin (H&E). The scoring system for total damage score was referenced from our previous study.^23^

### 2.4 Immunofluorescence and immunochemistry

Cell immunofluorescence: cells were fixed using 4% paraformaldehyde for 10 min, and washed with cold PBS. Cells were incubated with 0.1% Triton X‐100 in PBS for 15 min and washed with cold PBS. Cells were incubated with 5% bovine serum albumin in PBS for 1 h at room temperature (about 25°C). The primary antibodies against nuclear factor kappa‐B p65 (NF‐κB p65) and nuclear factor erythroid 2‐related factor 2 (Nrf2) (ABclonal) diluted to 1:500 were used to incubate the cells at 4°C overnight and washed with cold PBS. Next, cells were incubated with secondary antibodies (Thermo Fisher Scientific) (1:500) at room temperature for 1 h, followed by washing with cold PBS. Cells were staining with 4′,6‐diamidino‐2‐phenylindole for 5 min. Images were acquired using a Zeiss LSM 710 NLO Multiphoton microscope (Carl Zeiss).

Immunohistochemistry: immunohistochemistry was performed as our previous study.^24^ Primary antibodies zona occludens 1 (ZO‐1) (1:500; Proteintech), occludin (1:500; Abclonal), claudin 1 (1:500; Abclonal), mucin 2 (MUC2) (1:500; Proteintech), and mucin 3A (MUC3A) (1:500; Boster) were used to incubate the slides at 4°C overnight.

Mucicarmine staining: deparaffinating the sections with xylene and hydrating with different concentration of ethanol and water. Mayer hematoxylin was used to stain the sections for 10 min and rinse in running tap water for 2 min. Sections were dipped into bluing reagent for 30 s and rinsed in distilled water. Sections were stained with mucicarmine solution for 20 min and rinsed in tap water for 2 min followed by rinsing in distilled water. Sections were stained with tartrazine solution for 1 min and rinsed in running tap water. Next, dehydration in alcohol and transparency in xylene. Sealing the sections with synthetic resin.

### 2.5 Cell culture

RAW264.7 cells, a murine macrophage cell line, was purchased from BeNa Culture Collection, and cultured with Dulbecco's modified Eagle's medium (DMEM)/HIGH GLUCOSE (Hyclone) supplemented with 10% fetal bovine serum (FBS) (Gibco) and maintained at 37°C in a humidified chamber of 5% CO_2_. MCEC cells, a mouse colon epithelial cell line, was purchased from Bluefbio. The cells were cultured with DMEM/HIGH GLUCOSE (Hyclone) supplemented with 10% FBS (Gibco) and maintained at 37°C in a humidified chamber of 5% CO_2_.

### 2.6 Cell viability assay

Cell counting kit‐8 (CCK‐8) was used to analysis the effects of PD on the cell viability of RAW264.7 cells. Four different concentrations of PD (100, 200, 300, 400 μM) were used to treated the RAW264.7 cells. Twenty four hours later, 10 μl of CCK‐8 (Saint‐Bio) was used to treat for RAW264.7 cells for another one hour. Next, microplate reader was used to measure the OD value of RAW264.7 cells at 450 nm, each sample was analyzed in quintuplicate (*n* = 5).

### 2.7 Cell coculture

The cells were starved with FBS‐free DMEM/HIGH GLUCOSE culture medium for 3 h. PD (400 μM) was used to treat the cells for 1 h followed by treatment with 1 μg/ml LPS for 24 h. The supernatant was collected and centrifuged at 12,000*g* for 10 min. Next, collected the medium supernatant and used to treat MCEC cells. After 24 h, the MCEC cells were collected and total RNA was extracted, which was used to determine the expression of tight junction proteins, including Claudin‐1, occludin, and ZO‐1.

## 3 Reverse‐transcription polymerase chain reaction

Total RNA was extracted from RAW264.7 or MCEC cells using RNAiso Plus (Takara), and 0.5 μg RNA was used to generate complementary DNA using a commercial RT‐PCR kit (Takara). Next, reverse‐transcription polymerase chain reaction (RT‐PCR) was conducted using Hieff qPCR SYBR Green Master Mix (No Rox) (Yeasen), each sample was analyzed in triplicate. The sequences of primers used in this study are listed in Table 1.

**Table 1:** The sequence of primers used for RT‐PCR

**Gene**	**Primer sequence**	
*TNF‐α*	5′‐CCCCAAAGGGATGAGAAGTTC‐3′	5′‐CCTCCACTTGGTGGTTTGCT‐3′
*IL‐6*	5′‐CCAGAAACCGCTATGAAGTTCC‐3′	5′‐GTTGGGAGTGGTATCCTCTGTGA‐3′
*IL‐1β*	5′‐GTTCCCATTAGACAACTGCACTACAG‐3′	5′‐GTCGTTGCTTGGTTCTCCTTGTA‐3′
*iNOS*	5′‐GAACTGTAGCACAGCACAGGAAAT‐3′	5′‐CGTACCGGATGAGCTGTGAAT‐3′
*COX‐2*	5′‐CAGTTTATGTTGTCTGTCCAGAGTTTC‐3′	5′‐CCAGCACTTCACCCATCAGTT‐3′
*β‐actin*	5′‐GTCAGGTCATCACTATCGGCAAT‐3′	5′‐AGAGGTCTTTACGGATGTCAACGT‐3′
*ZO‐1*	5′‐GACCTTGATTTGCATGACGA‐3′	5′‐AGGACCGTGTAATGGCAGAC‐3′
*Claudin‐1*	5′‐AGGTCTGGCGACATTAGTGG‐3′	5′‐CGTGGTGTTGGGTAAGAGGT‐3′
*occludin*	5′‐ACACTTGCTTGGGACAGAGG‐3′	5′‐AAGGAAGCGATGAAGCAGAA‐3′
*β‐actin*	5′‐GTCAGGTCATCACTATCGGCAAT‐3′	5′‐AGAGGTCTTTACGGATGTCAACGT‐3′

Abbreviation: RT‐PCR, reverse‐transcription polymerase chain reaction.

### 3.1 Western blot

Protein was extracted from RAW264.7 cells using radio immunoprecipitation lysis buffer (Solarbio, China), centrifuging at 12,000*g* at 4°C for 15 min, and collecting the suspension liquid. The supernatant protein was quantified using a Pierce BCA protein Assay Kit (Thermo Fisher Scientific). The detail process referenced our previous study.^23^ Primary antibodies against p38, extracellular signal‐regulated kinase 1/2 (ERK1/2), c‐Jun N‐terminal kinase 1/2 (JNK1/2) and protein kinase B (AKT) (Santa Cruz), phosphorylation c‐Jun N‐terminal kinase 1/2 (p‐JNK1/2) (Cell Signaling Technology), phosphorylation protein kinase B (p‐AKT), p‐p38, phosphorylation extracellular signal‐regulated kinase 1/2 (p‐ERK1/2), inducible nitric oxide synthase (iNOS), Cyclooxygenase 2 (COX‐2) and NADPH Quinone acceptor Oxidoreductase 1 (NQO1) (ABclonal), Nrf2 and heme oxygenase‐1 (HO‐1) were diluted to 1:2000 to incubate the polyvinylidene difluoride membrane (Proteintech). Antibody against glyceraldehyde‐3‐phosphate dehydrogenase was diluted to 1:2000 (Absin). The secondary antibodies, goat against rabbit and goat against mice (Absin) were diluted to 1:10,000. Membranes were visualized by Amersham Imager 600 (a gel imaging system form GE Co.) after using enhanced chemiluminescence (ECL kit; Applygen Inst. Biotech). ImageJ software was used to numeralization the western blot results.

### 3.2 Statistical analysis

In this study, data showed as mean ± *SEM*. GraphPad Prism software version 7.00 was used for all statistical analysis. Groups were compared by one‐way analysis of variance followed by the least significant difference test (*p < 0.05, **p < 0.01, ***p < 0.001). Multiple comparison between the groups was performed using Tukey's test for post hoc analysis. Three replicates were conducted for each group in this study.

## 4 RESULTS

### 4.1 PD ameliorated weight loss, colon length, DAI, and intestine damage in DSS‐induced mice

**Figure 1:**
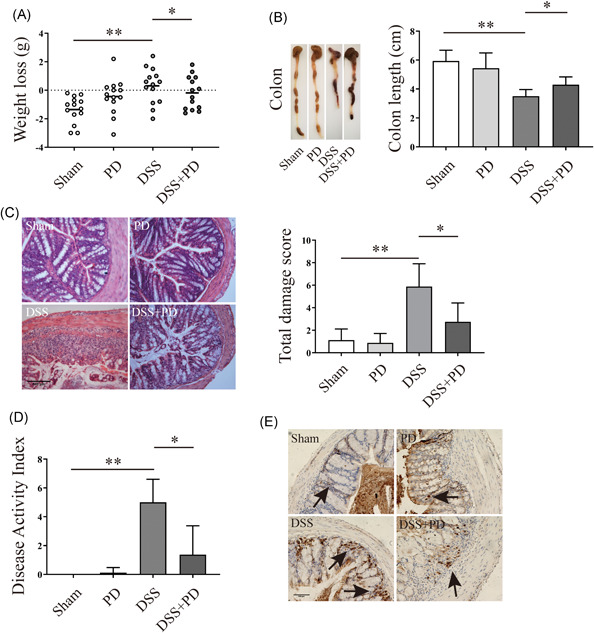
PD ameliorated weight loss, colon length, DAI, and intestine damage in DSS‐induced mice. (A) The weight loss (the initiation weight of mice—the weight of DSS treated for 6 days' mice) of PD or DSS treated mice (the mice number is 14, *n* = 14). (B) Colon length of PD or DSS treated mice (*n* = 14). (C) H&E staining analysis the damage score of PD or DSS treatment mice, magnification is ×20 (*n* ≥ 4). (D) DAI of PD or DSS treatment mice (*n* = 14). (E) Immunohistochemistry analysis the expression of MPO l, which representative the amount of neutrophil, black arrow mark the protein, magnification is ×20 (*n* ≥ 4). DAI, disease activity index; DSS, dextran sodium sulfate; H&E, hematoxylin and eosin; PD, polydatin. *p < 0.05, **p < 0.01

To evaluate the potential protective role of PD in DSS‐induced colitis mice, we observed their weight loss, DAI, colon length and intestine damage. The weight loss of the mice significantly increased 6 days after the initiation of DSS intake, whereas PD alleviated this tendency (Figure 1A). PD significantly increased the colon length in DSS‐induced mice (Figure 1B). To further evaluate the improvements of PD in DSS‐induced mice, we performed H&E staining. We found that DSS increased the damage score of mouse colons, whereas PD significantly decreased the damage score (Figure 1C). The protective role of PD was also observed in DAI in DSS‐induced mice (Figure 1D). DSS treatment facilitated the infiltration of neutrophil of the inflammatory area, whereas PD reduced the infiltration of neutrophil in DSS‐induced mice (Figure 1E). All results indicated that PD has a protective effects in DSS‐induced mice.

### 4.2 PD maintained proper intestinal epithelial barrier integrity in DSS‐induced mice

**Figure 2:**
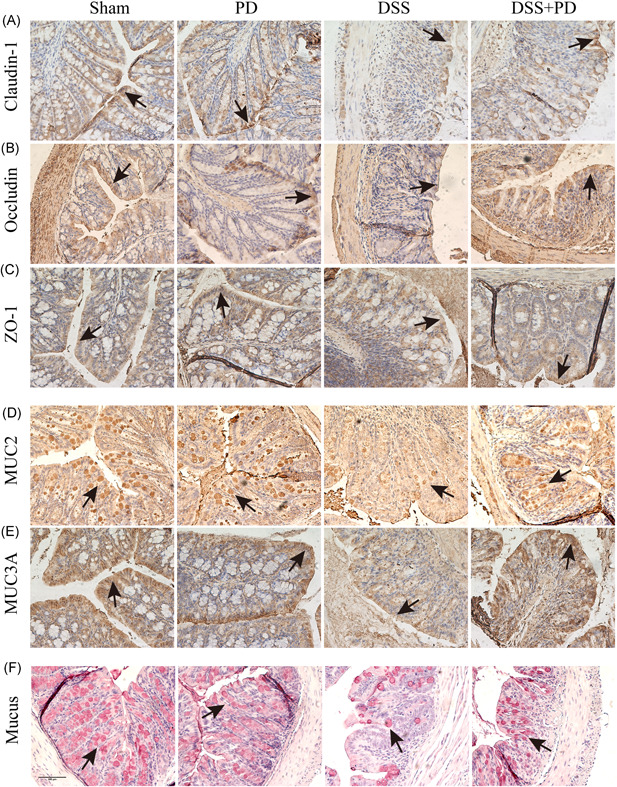
PD maintained proper intestinal epithelial barrier in DSS‐induced mice. (A–C) Immunohistochemistry analysis the expression of Claudin‐1, Occludin and ZO‐1 of colon tissue from DSS and PD treatment mice, black arrow mark the protein (*n* ≥ 4). (D, E) Immunohistochemistry analysis the expression of MUC2 and MUC3A in colon tissue from DSS and PD treatment mice, black arrow mark the protein (*n* ≥ 4). (F) Mucicarmine staining analysis the mucus in DSS and PD treatment mice colon tissue, magnification is ×20, black arrow mark the mucus (*n* ≥ 4). DSS, dextran sodium sulfate; PD, polydatin

To further evaluate the protective role of PD on IBDs, the intestinal epithelial barrier was measured during the experiments. Immunohistochemistry results showed that DSS decreased the expression of claudin‐1, occludin and ZO‐1, whereas PD improved the expression of claudin‐1, occludin and ZO‐1 in colon tissue (Figure 2A‐C). Our results revealed that DSS treatment decreased the secretion of MUC2 and the expression of MUC3A in the inflamed area of colon tissue; however, supplementation with PD markedly improved the amounts of MUC2 and MUC3A (Figure 2D,E). Substantial reduction of mucus in the colon 6 days after DSS intake, was also observed, and PD improved mucus in the colon tissue (Figure 2F). Taken together, our results revealed that DSS destroyed the intestinal epithelial barrier, whereas PD had a protective role on intestinal epithelial barrier integrity in DSS‐induced mice by maintaining the expression of the tight junction proteins, MUC2, MUC3A, and mucus.

### 4.3 PD suppressed LPS‐induced inflammation of macrophages

**Figure 3:**
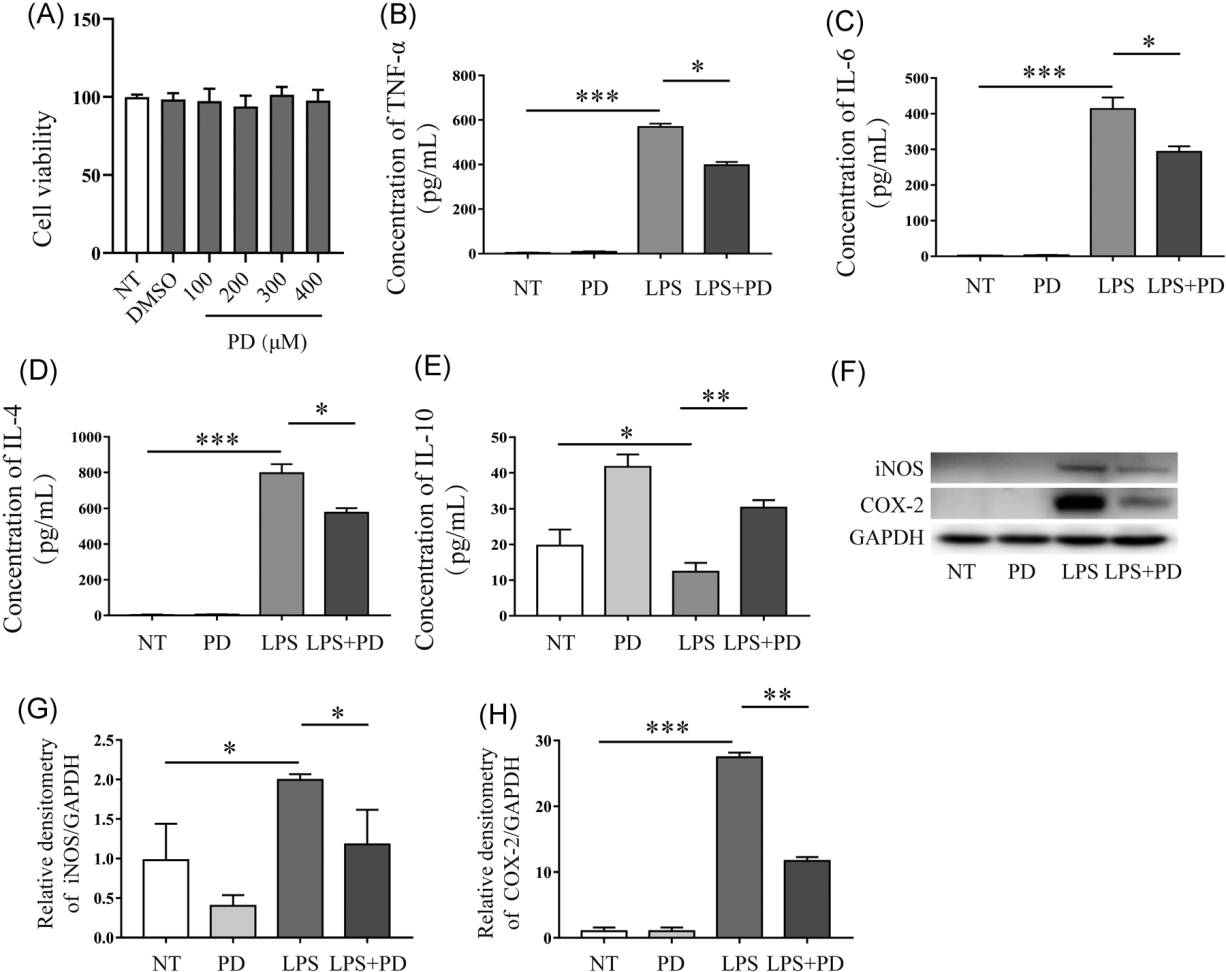
PD suppressed the inflammatory response in LPS‐induced macrophages. (A) Cell counting kit‐8 (CCK‐8) analyzed the effects of PD (100, 200, 300, and 400 μM) on the viability of RAW264.7 cells (the number of repetition is 5, *n* = 5). (B–E) RAW264.7 cells were treated using PD for 1 h followed by treatment with LPS (1 μg/ml) for 6 h. The level of (B) TNF‐α, (C) IL‐6, (D) IL‐4, (E) IL‐10 protein was determined by ELISA (*n* = 3). (F–H) RAW246.7 cells were treated using PD for 1 h followed by stimulating using LPS for 12 h, the protein was used to analyses the expression of iNOS and COX‐2 by Western blot (*n* = 3). COX‐2, cyclooxygenase‐2; ELISA, enzyme‐linked immunosorbent assay; IL, interleukin; iNOS, inducible nitric oxide synthase; LPS, lipopolysaccharide; PD, polydatin; TNF, tumor necrosis factor. *p < 0.05, **p < 0.01, and ***p < 0.001

**Figure 4:**
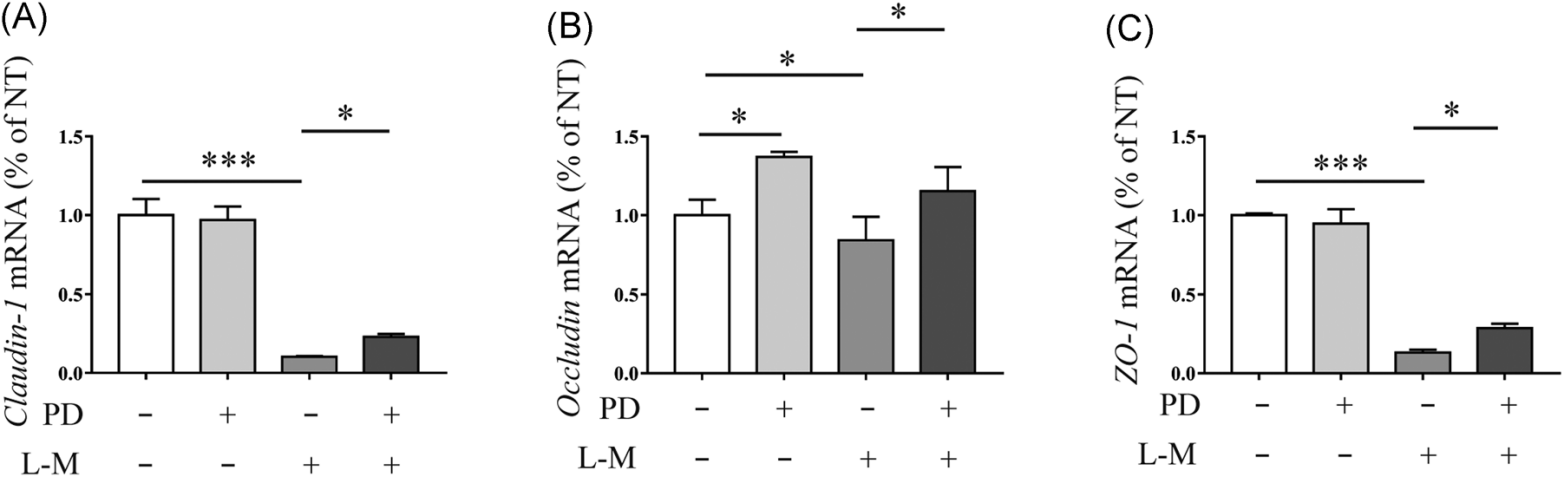
PD maintained intestinal epithelial barrier integrity by inhibiting the secretion of inflammatory cytokines. RAW264.7 cells were stimulate using LPS (1 μg/ml) for 12 h, next, the culture medium was collected and centrifuged, and used to treat the MCEC cells for 12 h. RT‐PCR was performed to analyses the level of (A) Claudin‐1, (B) Occludin, and (C) ZO‐1 mRNA in MCEC cells (*N* = 3). LPS, lipopolysaccharide; mRNA, messenger RNA; PD, polydatin; RT‐PCR, reverse transcription polymerase chain reaction. *p < 0.05, **p < 0.01, and ***p < 0.001

Cell viability assay showed that 100, 200, 300, and 400 μM of PD had no effect on cell viability, thus, 400 μM of PD was used to explore the anti‐inflammatory effects in LPS‐induced macrophages (Figure 3A). The results showed that LPS significantly increased the secretion of proinflammatory mediators TNF‐α, IL‐6, and IL‐4, whereas pretreatment with PD decreased the secretion of Pro‐inflammatory mediators (Figure 4B‐D). PD increased the secretion of IL‐10 in LPS‐induced macrophages (Figure 4E). LPS significantly increased the expression of iNOS and COX‐2, while supplementation with PD decreased the level of proinflammatory enzymes (Figure 4F‐H). All findings indicated that supplementation with PD inhibited the inflammatory response in LPS‐induced macrophages.

### 4.4 PD maintained intestinal epithelial barrier integrity by inhibiting the secretion of inflammatory cytokines

In our previous study, we had showed that the inhibition of proinflammatory mediator secretion is contributes to maintain proper expression of tight junction proteins in epithelial cells.^23^ In this study, the RT‐PCR results showed that the medium of LPS‐induced macrophages significantly decreased the level of ZO‐1, occludin and claudin‐1 messenger RNA in MCEC cells, while supplementation with PD improved the expression of these tight junction proteins (Figure 4A‐C). The results indicated that the protective function of PD on the intestinal epithelial barrier is associated with the inhibition of the inflammatory response.

### 4.5 The effects of PD on NF‐κB p65, AKT/NF‐κB/NQO‐2/HO‐1, and MAPKs signaling pathways

**Figure 5:**
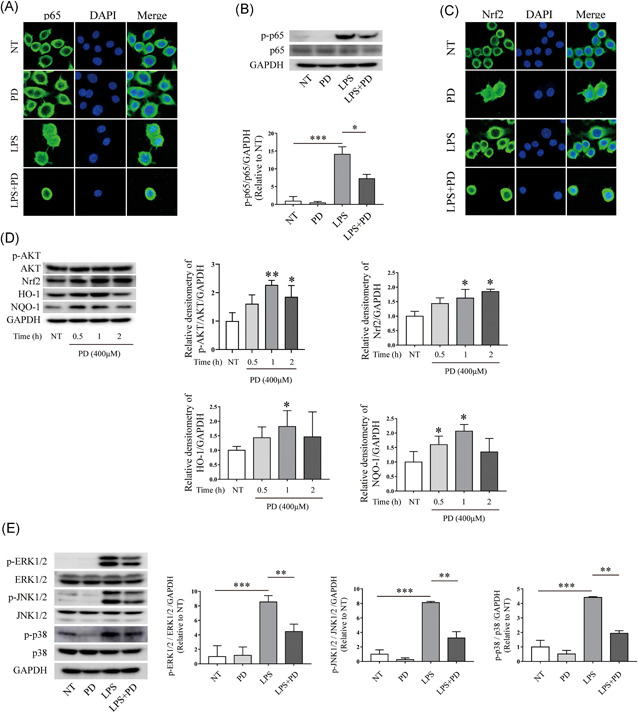
PD inhibited the phosphorylation and nuclear translocation of NF‐κB p65 signaling pathway. PD was used to treated RAW264.7 cells for 1 h followed by stimulating using LPS (1 μg/ml) for 1 h. (A) Cell immunofluorescence was performed to evaluate nuclear translocation of NF‐kB p65 signaling pathway, magnification is ×63 (*n* = 3). (B) After treatment with PD and LPS, Western blot was used to determine the phosphorylation of NF‐κB p65 signaling pathway (*n* = 3). (C) RAW264.7 cells were treated using PD for 1 h followed by stimulating using LPS (1 μg/ml) for 1 h. Cell immunofluorescence analyses the nuclear translocation of the Nrf2 signaling pathway, magnification is ×63 (*n* = 3). (D) RAW264.7 cells were stimulated with PD (400 μM) for 0.5, 1, and 2 h. Total protein was extracted from PD treated cells and used to determine the phosphorylation of AKT signaling pathway, and the expression level of Nrf2, HO‐1, and NQO‐1 (*n* = 3). (E) PD was used to treat RAW264.7 cells 1 h followed by treating using LPS (1 μg/ml) for 1 h. Protein was extracted from RAW264 cells and used to determine the phosphorylation of ERK1/2, JNK1/2, and p38 signaling pathway. PD treatment inhibited the phosphorylation of ERK1/2, JNK1/2, and p38 signaling pathway (*n* = 3). ERK, extracellular signal‐regulated kinase; JNK, c‐Jun N‐terminal kinase; LPS, lipopolysaccharide; NF‐κB, nuclear factor‐κB; PD, polydatin. *p < 0.05, **p < 0.01, and ***p < 0.001

The phosphorylation and nuclear translocation of the NF‐κB p65 signaling pathway are associated with an inflammatory response. Therefore, we measured the effects of PD on the phosphorylation and nuclear translocation of NF‐κB p65 signaling pathway. Results showed that LPS sharply increased the phosphorylation and accelerated the nuclear translocation of the NF‐κB p65 signaling pathway, while PD blocked its phosphorylation and inhibited its nuclear translocation (Figure 5A,B). Oxidative stress is often observed in the inflamed intestinal mucosa and deeper layers of the intestinal wall in IBD.^25^ Nrf2 is the most important transcription factor that against oxidative stress and plays a crucial role in antioxidants and relieve inflammation.^26^ In response to oxidative stress Nrf2 initiates an antioxidant response by activating the expression of NQO‐1 and HO‐1. The activation of Nrf2/NQO‐1 redox pathway significantly reduce the inflammation in DSS‐induced mice.^27^ Therefore, we next evaluated the effects of PD on Nrf2/NQO‐1/HO‐1 in RAW264.7 macrophages. Our results showed that PD accelerated the nuclear translocation of Nrf2 (Figure 5C), and PD treatment improved the expression of Nrf2 (Figure 5D). PD treatment increased the expression of NQO‐1 and HO‐1 (Figure 5D). The upregulation of Nrf2 depend on the activation of AKT and the inactivation GSK‐3β.^28^ Therefore, we determined the variation of AKT after treatment with PD and found that it significantly increased the phosphorylation of the AKT signaling pathway in macrophages (Figure 5D). The results showed that PD may provide an antioxidant effect in RAW264.7 macrophages. Mitogen‐activated protein kinase (MAPK) signaling pathways can be activated in response to a diverse array of stimulating factors, such as LPS, subsequently regulate cell inflammation. Therefore, MAPK can be used for the targets for development of novel anti‐inflammatory drugs.^29^ In this study, we also determined the effects of PD on MAPK signaling pathway in LPS‐induced macrophages. Results showed that LPS sharply accelerated the phosphorylation of the ERK1/2, JNK1/2 and p38 signaling pathways (Figure 5E), whereas supplementation using PD inhibited the phosphorylation of ERK1/2, JNK1/2, and p38 (Figure 5E) signaling pathways.

## 5 DISCUSSION

UC is a complex and refractory intestinal inflammatory disease that is characterized by long‐lasting mucosal inflammation, initiating in the rectum and extending to proximally of colon in a continuous fashion.^30^ In UC, inflammation is restricted to the mucosal layer of the intestine, resulting in ulceration and bloody diarrhea.^31^ As DSS‐induced colitis mice exhibit similar clinical manifestations, they are regarded one of the most successful and appropriate colitis models, and are widely used to study the function of new drugs, extracts, and single compounds and mechanistic studies of colitis.^32^ Therefore, in the present study, we used DSS‐induced colitis to explore the protective effects of PD, and explored its mechanism in RAW264.7 macrophages.

In this study, the improvement effects of PD in DSS‐induced mice were assessed by a series of indices, such as weight loss, colon length, DAI, colitis score, mucin, and tight junction protein deletion. The loss of body weight, shortening of the colon length, and increasing of DAI are classical clinical symptoms that are widely used to reflect the severity and prognosis of DSS‐induced colitis.^33^ Here, we found that PD decreased weight loss, increased colon length, and decreased DAI in DSS‐induced colitis mice. PD reduced the intestinal inflammatory response, as assessed by the histological score (H&E staining), and decreased the infiltration of neutrophil in DSS‐induced mice colon tissue. UC is an intestinal barrier disease initially caused by the dysfunction of epithelial cells or structural intestinal epithelial.^34^ The impaired intestinal epithelium barrier allows antigen, exotoxin, and other exogenous substance to enter, which activate macrophages, antigen‐presenting cells, natural killer cells and neutrophils. Subsequently, the levels of the proinflammatory cytokines TNF, IL‐9, IL‐13, IL‐23, and IL‐36, which are key cytokines that drive the inflammatory cascade, increase, thereby leading to the chronicity of UC. Furthermore, these proinflammatory cytokines alter the expression level of tight junction proteins, further leading to impair the intestinal epithelium barrier and allowing more exogenous substances to cross the epithelial barrier.^31^ Traditional drugs, biologic therapies, and recently emergent small molecular inhibitors all directly inhibit the inflammatory response. Therefore, inhibition of the intestinal inflammatory response is a critical therapy for UC. Our previous study indicated that the inhibition of proinflammatory cytokines in macrophages maintains the expression level of tight junction proteins.^23^ In the present study, we found that PD suppressed inflammation in DSS‐induced mice and maintained the expression of tight junction proteins, mucin proteins, and mucus.

The augmented production of proinflammatory cytokines, such as TNF‐α, IL‐1β, IL‐6, IL‐18, and interferon‐γ, was observed in colon of DSS‐induced mice.^35^ proinflammatory cytokines accelerate the pathogenesis of IBD by impairing the intestinal epithelium barrier and facilitating the inflammatory response. LPS is a cell‐associated glycolipid from the Gram‐negative bacteria that activates innate immune cells and promotes the expression of proinflammatory cytokines by stimulating signaling through toll‐like receptor 4.^36^ In this study, we found that PD significantly inhibited the expression of TNF‐α, IL‐4, and IL‐6 in LPS‐induced macrophages, and increased the expression of IL‐10. The reduction of proinflammatory cytokines helped maintain the epithelial barrier. PD increased the expression of ZO‐1, claudin‐1, and occludin in medium (LPS‐induced macrophages medium)‐induced MCEC cells. Taken together, our results showed that PD inhibited the inflammatory response and provided a protective role in maintain intestinal epithelium barrier integrity.

MAPKs contains three major protein kinases, including the ERK, JAKs, and p38 MAPKs. MAPKs associate with various transcription factors in IBD. NF‐κB is a well‐studied transcription factor of MAPK downstream signaling. P38 MAPK promote the phosphorylation of NF‐κB p65 through mitogen‐ and stress‐activated kinase 1.^37^ NF‐κB is also implicated in patients with IBD patients and colitis mice, and promotes the secretion of pro‐inflammatory cytokines, such as TNF‐α, IL‐1, IL‐2, IL‐6, IL‐8, and IL‐12.^38^ In this study, we found that LPS significantly improved the nuclear translocation and the phosphorylation of NF‐κB p65, promote the phosphorylation of Erk1/2, JNK1/2, and p38 signaling pathway, whereas PD significantly reduced the tendency.

In IBD, oxidative stress observed in the inflamed area of mucosa and the deeper layers of the intestinal wall.^25^ Nrf2 is the most important transcription factor which against oxidative stress and plays a crucial role in antioxidants and relieve inflammation.^26^ After oxidative stress, Nrf2 escapes from Keap 1 and translocates into the nucleus to activate antioxidant enzymes, including HO‐1, NQO‐1, glutathione peroxidase and glutamate‐cysteine ligase.^39^ In this study, PD promoted the nuclear transcription of Nrf2, and increased the expression of Nrf2, HO‐1, and NQO‐1. It is indicated that PD have antioxidant effects in DSS‐induced mice, which is in accordance with the results of previous study.^21^

## 6 CONCLUSION

In summary, our results indicated that PD maintains the epithelial barrier by inhibiting intestinal inflammation, and the mechanism of the anti‐inflammatory effects is likely through the inhibition of MAPKs, the NF‐κB inflammation signaling pathway, and the AKT/Nrf2/HO‐1/NQO1 mediated antioxidant signaling pathway.

## CONFLICT OF INTEREST

The authors declare that there are no conflict of interests.

## AUTHOR CONTRIBUTIONS

Lina Dong contributed to conceptualization and data curation. Guangxin Chen contributed to funding acquisition, main investigation, and writing–original draft. Ziyue Yang, Da Wen, Jian Guo, Qiuhong Xiong, and Liping Zhao contributed to investigation, including immunohistochemistry, Mayer's carmine staining, and Western blot. Junping Wang, Changxin Wu, and Lina Dong completed Review and editinr. All authors have read and agreed to the published version of the manuscript.

## Data Availability

The data that support the findings of this study are available from the corresponding author upon reasonable request.

## References

[1] Honda K , Littman DR . The microbiota in adaptive immune homeostasis and disease. Nature. 2016;535:75‐84. 27383982 10.1038/nature18848

[2] Baumgart DC , Sandborn WJ . Crohn's disease. Lancet. 2012;380:1590‐1605. 22914295 10.1016/S0140-6736(12)60026-9

[3] Graham DB , Xavier RJ . Pathway paradigms revealed from the genetics of inflammatory bowel disease. Nature. 2020;578:527‐539. 32103191 10.1038/s41586-020-2025-2 PMC7871366

[4] Ananthakrishnan AN . Epidemiology and risk factors for IBD. Nat Rev Gastroenterol Hepatol. 2015;12:205‐217. 25732745 10.1038/nrgastro.2015.34

[5] Ng SC , Shi HY , Hamidi N , et al. Worldwide incidence and prevalence of inflammatory bowel disease in the 21st century: a systematic review of population‐based studies. Lancet. 2018;390:2769‐2778. 10.1016/S0140-6736(17)32448-0 29050646

[6] Pouillon L , Travis S , Bossuyt P , Danese S , Peyrin‐Biroulet L . Head‐to‐head trials in inflammatory bowel disease: past, present and future. Nat Rev Gastroenterol Hepatol. 2020;17:365‐376. 32303700 10.1038/s41575-020-0293-9

[7] Ho GT , Cartwright JA , Thompson EJ , Bain CC , Rossi AG . Resolution of inflammation and gut repair in IBD: translational steps towards complete mucosal healing. Inflamm Bowel Dis. 2020;26:1131‐1143. 32232386 10.1093/ibd/izaa045 PMC7365805

[8] Soendergaard C , Bergenheim FH , Bjerrum JT , Nielsen OH . Targeting JAK‐STAT signal transduction in IBD. Pharmacol Ther. 2018;192:100‐111. 30048708 10.1016/j.pharmthera.2018.07.003

[9] Neurath MF . New targets for mucosal healing and therapy in inflammatory bowel diseases. Mucosal Immunol. 2014;7:6‐19. 24084775 10.1038/mi.2013.73

[10] Ahmad P , Alvi SS , Iqbal D , Khan MS . Insights into pharmacological mechanisms of polydatin in targeting risk factors‐mediated atherosclerosis. Life Sci. 2020;254:117756. 32389832 10.1016/j.lfs.2020.117756

[11] Chen P , Wang L , Sun S , et al. High‐throughput screening suggests glutathione synthetase as an anti‐tumor target of polydatin using human proteome chip. Int J Biol Macromol. 2020;161:1230‐1239. 32544581 10.1016/j.ijbiomac.2020.06.061

[12] Sun Z , Wang X . Protective effects of polydatin on multiple organ ischemia‐reperfusion injury. Bioorg Chem. 2020;94:103485. 31836186 10.1016/j.bioorg.2019.103485

[13] Gao Y , Dai X , Li Y , et al. Role of Parkin‐mediated mitophagy in the protective effect of polydatin in sepsis‐induced acute kidney injury. J Transl Med. 2020;18:114. 32131850 10.1186/s12967-020-02283-2 PMC7055075

[14] Chen X , Chan H , Zhang L , et al. The phytochemical polydatin ameliorates non‐alcoholic steatohepatitis by restoring lysosomal function and autophagic flux. J Cell Mol Med. 2019;23:4290‐4300. 30973211 10.1111/jcmm.14320 PMC6533566

[15] Huang QH , Xu LQ , Liu YH , et al. Polydatin Protects Rat Liver against ethanol‐induced injury: involvement of CYP2E1/ROS/Nrf2 and TLR4/NF‐kappaB p65 pathway. Evid Based Complement Alternat Med. 2017;2017:7953850. 29250126 10.1155/2017/7953850 PMC5698823

[16] Huang B , Liu J , Meng T , et al. Polydatin prevents lipopolysaccharide (LPS)‐induced Parkinson's disease via regulation of the AKT/GSK3beta‐Nrf2/NF‐kappaB signaling axis. Front Immunol. 2018;9:2527. 30455692 10.3389/fimmu.2018.02527 PMC6230593

[17] Peng Y , Xu J , Zeng Y , Chen L , Xu XL . Polydatin attenuates atherosclerosis in apolipoprotein E‐deficient mice: Role of reverse cholesterol transport. Phytomedicine. 2019;62:152935. 31085374 10.1016/j.phymed.2019.152935

[18] Bheereddy P , Yerra VG , Kalvala AK , Sherkhane B , Kumar A . SIRT1 activation by polydatin alleviates oxidative damage and elevates mitochondrial biogenesis in experimental diabetic neuropathy. Cell Mol Neurobiol. 2020 10.1007/s10571-020-00923-1 PMC11448605 32683581

[19] Gugliandolo E , Fusco R , Biundo F , et al. Palmitoylethanolamide and Polydatin combination reduces inflammation and oxidative stress in vascular injury. Pharmacol Res. 2017;123:83‐92. 28676456 10.1016/j.phrs.2017.06.014

[20] Hu T , Fei Z , Su H , Xie R , Chen L . Polydatin inhibits proliferation and promotes apoptosis of doxorubicin‐resistant osteosarcoma through LncRNA TUG1 mediated suppression of Akt signaling. Toxicol Appl Pharmacol. 2019;371:55‐62. 30974157 10.1016/j.taap.2019.04.005

[21] Lv T , Shen L , Yang L , et al. Polydatin ameliorates dextran sulfate sodium‐induced colitis by decreasing oxidative stress and apoptosis partially via Sonic hedgehog signaling pathway. Int Immunopharmacol. 2018;64:256‐263. 30218952 10.1016/j.intimp.2018.09.009

[22] Murano M , Maemura K , Hirata I , et al. Therapeutic effect of intracolonically administered nuclear factor kappa B (p65) antisense oligonucleotide on mouse dextran sulphate sodium (DSS)‐induced colitis. Clin Exp Immunol. 2000;120:51‐58. 10759763 10.1046/j.1365-2249.2000.01183.x PMC1905625

[23] Chen G , Ran X , Li B , et al. Sodium butyrate inhibits inflammation and maintains epithelium barrier integrity in a TNBS‐induced inflammatory bowel disease mice model. EBioMedicine. 2018;30:317‐325. 29627390 10.1016/j.ebiom.2018.03.030 PMC5952406

[24] Chen G , Liu J , Jiang L , et al. Galangin reduces the loss of dopaminergic neurons in an LPS‐evoked model of Parkinson's disease in rats. Int J Mol Sci. 2017;19. 10.3390/ijms19010012 PMC5795964 29267220

[25] Moura FA , de Andrade KQ , Dos Santos JCF , Araujo ORP , Goulart MOF . Antioxidant therapy for treatment of inflammatory bowel disease: does it work? Redox Biol. 2015;6:617‐639. 26520808 10.1016/j.redox.2015.10.006 PMC4637335

[26] Kobayashi E , Suzuki T , Yamamoto M . Roles nrf2 plays in myeloid cells and related disorders. Oxid Med Cell Longev. 2013;2013:529219. 23819012 10.1155/2013/529219 PMC3684031

[27] Wang K , Lv Q , Miao YM , Qiao SM , Dai Y , Wei ZF . Cardamonin, a natural flavone, alleviates inflammatory bowel disease by the inhibition of NLRP3 inflammasome activation via an AhR/Nrf2/NQO1 pathway. Biochem Pharmacol. 2018;155:494‐509. 30071202 10.1016/j.bcp.2018.07.039

[28] Xin Y , Bai Y , Jiang X , et al. Sulforaphane prevents angiotensin II‐induced cardiomyopathy by activation of Nrf2 via stimulating the Akt/GSK‐3ss/Fyn pathway. Redox Biol. 2018;15:405‐417. 29353218 10.1016/j.redox.2017.12.016 PMC5975128

[29] Karin M . Mitogen activated protein kinases as targets for development of novel anti‐inflammatory drugs. Ann Rheum Dis 63. 2004;Suppl 2:ii62‐ii64. 10.1136/ard.2004.028274 PMC1766783 15479874

[30] Rubin DT , Ananthakrishnan AN , Siegel CA , Sauer BG , Long MD . ACG clinical guideline: ulcerative colitis in adults. Am J Gastroenterol. 2019;114:384‐413. 30840605 10.14309/ajg.0000000000000152

[31] NIH . Ulcerative colitis. *Nat Rev Dis Primers*, 2020, 6 (1):73. 10.1038/s41572-020-00215-4 32913215

[32] Chassaing B , Aitken JD , Malleshappa M , Vijay‐Kumar M . Dextran sulfate sodium (DSS)‐induced colitis in mice. Curr Protoc Immunol. 2014;104:15 25 11‐15 25 14. 24510619 10.1002/0471142735.im1525s104 PMC3980572

[33] Eichele DD , Kharbanda KK . Dextran sodium sulfate colitis murine model: An indispensable tool for advancing our understanding of inflammatory bowel diseases pathogenesis. World J Gastroenterol. 2017;23:6016‐6029. 28970718 10.3748/wjg.v23.i33.6016 PMC5597494

[34] Kobayashi T , Siegmund B , Le Berre C , et al. Ulcerative colitis. Nat Rev Dis Primers. 2020;6:74. 32913180 10.1038/s41572-020-0205-x

[35] Liang J , Chen S , Chen J , et al. Therapeutic roles of polysaccharides from Dendrobium Officinaleon colitis and its underlying mechanisms. Carbohydr Polym. 2018;185:159‐168. 29421053 10.1016/j.carbpol.2018.01.013

[36] Mancuso G , Midiri A , Biondo C , et al. Bacteroides fragilis‐derived lipopolysaccharide produces cell activation and lethal toxicity via toll‐like receptor 4. Infect Immun. 2005;73:5620‐5627. 16113279 10.1128/IAI.73.9.5620-5627.2005 PMC1231095

[37] Vermeulen L , De Wilde G , Van Damme P , Vanden Berghe W , Haegeman G . Transcriptional activation of the NF‐kappaB p65 subunit by mitogen‐ and stress‐activated protein kinase‐1 (MSK1). EMBO J. 2003;22:1313‐1324. 12628924 10.1093/emboj/cdg139 PMC151081

[38] Pasparakis M . Regulation of tissue homeostasis by NF‐kappaB signalling: implications for inflammatory diseases. Nat Rev Immunol. 2009;9:778‐788. 19855404 10.1038/nri2655

[39] Baird L , Dinkova‐Kostova AT . The cytoprotective role of the Keap1‐Nrf2 pathway. Arch Toxicol. 2011;85:241‐272. 21365312 10.1007/s00204-011-0674-5

